# A Temperature Conditioned Markov Chain Model for Predicting the Dynamics of Mosquito Vectors of Disease

**DOI:** 10.3390/insects12080725

**Published:** 2021-08-13

**Authors:** Petros T. Damos, Jesse Dorrestijn, Thomas Thomidis, José Tuells, Pablo Caballero

**Affiliations:** 1 Department of Community Nursing, Preventive Medicine, Public Health and History of Science, Faculty of Health Science, University of Alicante, Carretera San Vicente s/n, 03690 San Vicente del Raispeig, ALC, Spain; tuells@ua.es (J.T.); pablo.caballero@ua.es (P.C.); 2 Pharmacy Department, University General Infectious Disease Hospital of Thessaloniki AHEPA, Aristotle University of Thessaloniki, 54136 Thessaloniki, Greece; 3 Department of Nutritional Sciences and Dietetics, International Hellenic University of Thessaloniki, 57400 Thessaloniki, Greece; thomidis@cp.teithe.gr; 4 Faculty of Civil Engineering and Geoscience, Delft University of Technology, 2628 CN Delft, The Netherlands; jessedorrestijn2@gmail.com

**Keywords:** *Culex* sp., decision making, mosquitos, public health, stochastic process, West Nile virus

## Abstract

**Simple Summary:**

Understanding and predicting vector population and related disease dynamics, is crucial for gaining insight into the abundance and dynamics of arthropod disease vectors, and for the design of effective vector control strategies. Several mathematical and standard epidemiological models have been proposed in studying vector transmitted infectious disease dynamics. However, most models are of deterministic nature and are not able to estimate other relevant metrics such as the probability of vector population emergence as well as the probability and expected time to reach certain population and/or infection state. Here we are focusing on stochastic modeling of mosquito abundance data using weather driven Markov chains (MCs) and are particularly interested in estimating transition probabilities (TPs) between different population levels. A MC model is based on the assumption that the future state of the variable is only dependent on the present state and is suitable in cases of short and noisy data characterized by a complex and random behavior. The aim is to introduce and generalize a formulation of conditional Markov chain models (CMSs) for predicting probability transition estimates of arthropod vector populations. In this context, first we present the basic principles and assumptions behind Markov chain modeling approach, with an intuitive interpretation of the integration of conditional Markov chains (CMCs) and then demonstrate the usefulness of the approach in predicting the abundance of *Culex* *sp*. We conclude that the conditional Markov chain technique is recommended as viable for modeling populations that explicit random dynamics and predict their future evolution. Although, the Markov models generated in this work provide an accurate abstraction of the vector disease progress observed within the dataset used for their generation, we envision the current approach as an entry point into the medical entomology literature and methods for predicting arthropod vector diseases dynamics.

**Abstract:**

Understanding and predicting mosquito population dynamics is crucial for gaining insight into the abundance of arthropod disease vectors and for the design of effective vector control strategies. In this work, a climate-conditioned Markov chain (CMC) model was developed and applied for the first time to predict the dynamics of vectors of important medical diseases. Temporal changes in mosquito population profiles were generated to simulate the probabilities of a high population impact. The simulated transition probabilities of the mosquito populations achieved from the trained model are very near to the observed data transitions that have been used to parameterize and validate the model. Thus, the CMC model satisfactorily describes the temporal evolution of the mosquito population process. In general, our numerical results, when temperature is considered as the driver of change, indicate that it is more likely for the population system to move into a state of high population level when the former is a state of a lower population level than the opposite. Field data on frequencies of successive mosquito population levels, which were not used for the data inferred MC modeling, were assembled to obtain an empirical intensity transition matrix and the frequencies observed. Our findings match to a certain degree the empirical results in which the probabilities follow analogous patterns while no significant differences were observed between the transition matrices of the CMC model and the validation data (ChiSq = 14.58013, df = 24, *p* = 0.9324451). The proposed modeling approach is a valuable eco-epidemiological study. Moreover, compared to traditional Markov chains, the benefit of the current CMC model is that it takes into account the stochastic conditional properties of ecological-related climate variables. The current modeling approach could save costs and time in establishing vector eradication programs and mosquito surveillance programs.

## 1. Introduction

Europe is confronting a rising threat of outbreaks of arthropod vector borne (AVB) tropical diseases as rising temperatures linked to climate change create a conducive environment for arthropod development and dispersion [1]. Mosquitos, among others, are vectors of many viruses and parasitic pathogens. They can carry diseases such as malaria or yellow fever and traditionally most of these pathogens are found in Africa, Asia and Latin America. However, the expansion of arthropods vectors (AVs) in temperate climates is now a direct consequence of the increased mobility of people in the era of globalization [1,2]. Most importantly, climate change has created conditions conducive to maintaining and developing vector mosquitoes in new areas. This leads to an increase in mosquito populations and the potential for virus transmission [3,4].

In addition to endemic mosquito species that are present in Europe and particularly Greece, such as those belonging to the genera *Culex*, *Anopheles* and *Aedes*, climate change and particularly the increase in average temperatures is expected to bring about extension to wider geographical units of Europe of more arthropod vector related diseases [5,6,7]. Over the last ten years, worldwide climatic conditions, including the Mediterranean and Greece, have encouraged the development of the mosquito population and particularly the transmission of West Nile virus (WNV) [8,9,10]. The primary reservoir of the virus in nature is mainly wild birds, from which mosquitoes are infected, while humans do not further transfer the virus to other mosquitoes [11,12,13]. The majority of people infected with the virus are asymptomatic, around 20% have mild symptoms of viral syndrome and less than 1% has more serious central nervous system manifestations, mainly encephalitis and meningitis. The most severe events usually occur in the elderly, immunocompromised patients and, in general, individuals with underlying chronic diseases [14].

In fact, vector-borne diseases are becoming a major threat, impacting both human and animal health with severe consequences in the governance of health risks attributed to these emerging diseases in European countries. Primary strategies focus on preventing human exposure by effective insect control. This work considers in mosquito-borne diseases and mostly those transmitted by *Culex* sp. and how they are affected by weather variables. One of the most effective ways to achieve this goal is based on prioritizing efforts in identifying what considerations should be taken into account to guide decisions such as variable weather in itself (e.g., drought, extreme temperatures) that could be detrimental to mosquito survival [15]. Arthropods are very sensitive to weather and therefore ongoing climatic trends of warming and more variable weather threaten to increase burden of these diseases [16]. In recent years, progressions in Earth observation satellites together with geographical information systems have contributed to weather monitoring. Mathematical models have become a valuable tool for predicting dynamics of populations under climatic scenarios and different temperature regimes improving our knowledge about contribution of environmental and biodiversity factors in vector-borne diseases and helping public health decision making for better allocation of resources in the fight against many pathogens [17].

Arthropod vector control, which relies on the use of insecticides, is the principal means of mitigating the spread of related diseases [18,19]. However, for such a strategy to be effective, it is important to predict the temporal change in mosquito abundance and how it is affected by weather conditions [20,21]. This is particularly relevant for epidemiological studies with arthropod vectors since their development and population dynamic are strongly affected by climatic conditions, changing conditions in a context of climate change.

For instance, weather conditions have direct and indirect effects on growth and development of mosquitoes. Additionally, gonotrophic activity by *Culex sp*. in early spring and during the season is affected by water resources and this establishes the subsequent phenology and determines the potential date of the earliest mosquito-borne encephalitis virus transmission [21]. Traditionally, the effect temperature over insect growth rate is captured by empirical linear and/or parabolic functions [22]. However, most often temperature-dependent population growth is determined by temporally fluctuating vital rates for which classical demographic theory often does not apply [23]. Moreover, actual mosquito flight patterns might be related to the extent of which blood-feeding behavior are shaped by available feeding resources [24]. As a result, in field conditions mosquito dynamics may be complex and characterized by abrupt outbreaks and overlapping generations. From an applied perspective, understanding adult mosquito flight patterns and how it is affected by weather is essential for predicting their activity to prevent the risk of transmitting related vector-borne pathogens in nature [25]. Identification, of periods of high population activity can guide effective targeting of the species. Consequently, several mathematical models have been used to connect the biological processes of vector dynamics and climate [26,27,28,29]. To date, most epidemiological and insect population models, have a deterministic nature and rely on some basic assumptions to define the various parameters of vector and disease dynamics under study [11,30]. Often these parameters are unknown and need first to be estimated to parameterize the model. Moreover, because of the impacts of various internal and external factors, the temporal evolution of population processes is non-linear and characterized by random perturbations making it difficult to analyze and forecast population dynamics using only empirical-temperature dependent growth and mechanistic models [31].

Stochastic models, which recognize that all variables are probabilistic in nature and are handled as such, could be employed to model non-linear ecological processes and advance our understanding of vector population dynamics for public health planning [31]. Markov chain (MC) models belong to the class of stochastic models in probability theory that are based on the Markov property, which assumes that future states of a process that evolves in time depend only on the current state and not on the events that occurred before [31]. Such a framework provides a coherent approach to solving and inferring practical issues of decision-making since it integrates multiple sources of uncertain information in a probabilistic way, and which often characterizes noisy stochastic processes [31,32,33].

Markov chain models have proved suitable for describing randomly changing systems such as queuing [34] and manufacturing systems [35], market trend analysis [36,37] and insurance methods [38,39]. Recently, MCs were used in modeling biological processes and health systems, such carcinogenesis [40,41] and medical cost health problems [42]. Applications of MCs in modeling categorical data sequences can also be found [43,44,45,46], including air population modeling [47], and weather forecasting [48,49], although there are fewer examples analyzing ecological time series and population dynamics [32,50].

To date, in the field of theoretical population biology, the concept of MCs can be associated to any to the projection of the Leslie matrix that contains information about its genealogy, and which can be transformed exactly into a Markov chain [51]. Additionally, developmental stage transition models have been formulated to estimate the transition from one stage to another and under different environmental conditions [52,53]. Most of the age-structured matrix projection models have been used in demographic studies as a central tool to quantify the asymptotic growth rates (i.e., the dominant eigenvalue) and the reproductive values (dominant right and left eigenvalues). They have been also used in the context of MCs to estimate the stable age distribution and how it is affected under certain environments [53,54].

However, very few studies have emphasized the application of MCs in studying mosquito dynamics and the effect of detrimental weather variables. Chaves et al. 2014 [24], for instance, has used a two-stage (larvae and adults) recruiting matrix model to propose a mechanism for environmental signal canalization into demographic parameters of *Aedes aegypti* that could explain delayed high temperature induced mosquito outbreaks. However, to our best knowledge, Markov chains have not been applied to solve the problem of predicting and simulating the probability of *Culex sp*. ecological time series and especially with respect to exogenous stochastic factors such as weather variables and particularly temperature. Hence, it is assumed that a temperature-conditioned Markov chain (CMC) model could be developed and applied for the first time to predict the dynamics of *Culex* sp. vectors of important medical diseases. The major advantage of the CMC over traditional Markov chain models is that via appropriate conditioning their primary Markov chain properties are mixed with that of relevant climate factors [55]. Additionally, the mosquito dynamics process is considered as random, in which no prior information of the system properties is needed, and thus the resultant dynamic stochastic model is purely data driven. It could be beneficial to develop new stochastic population modeling approaches that take into account the effect of one exogenous stochastic variable over the other by terms of conditional probabilities.

In addition, due to their complicated life cycle and overlapping of generations, mosquito populations are characterized by abrupt dynamics and thus cannot be easily predicted by traditional insect population models. Moreover, considering that most of their attributes can change in respect to random climate events, stochastic models become a suitable alternative candidate for describing and predicting their abundance [3,4,11,17].

In previous works [56] it was shown that the relationship between climate factors and mosquito abundance is not linear over the full data length, and that mosquito populations exhibit a high degree of non-linear behavior under field conditions. As a result, periods of mosquito growth and different population levels are interrupted by the presence of unfavorable temperature conditions in a random way. To date, despite Markov chain models being used extensively in turbulence and predictability studies, as well as disease dynamics, they have not been used to model the abundance of *Culex sp*.

In this context, the major objective of the current work was the development of a weather driven Markov chain model for simulating and predicting the population dynamics of arthropod vector dynamics. Moreover, the model is applied and tested on *Culex sp*. Mosquitos, which is the main vector of WNV transmission and thus of high medical importance. Additionally, the aim is to contribute to a precise prediction of the adult mosquito dynamics through the application of a conditional Markov chains. This paper presents a general stochastic modeling approach enabling the multivariate analysis of *Culex sp*. population dynamics and related weather variables. Avoiding inherent relationship assumptions and parameter estimation in deterministic models; stochastic models provide a realistic data-based alternative to simulation of complex systems and robust predictions that could make informed and sound decisions.

In the next section, we show the model derivation and final formulation, while in Section 3 we apply the model for predicting *Culex sp*. first with real data and numerical simulations of the population dynamics of mosquitos, then conditioned by the most detrimental weather variable [56]. Additionally, we made efforts to validate the model using empirical data and which have not been used to parameterize the models. In the end, we briefly discuss the modeling, prediction results of the current subject field, as well as over the pros and cons of the proposed mathematical modeling approach and how it can lead to our understanding of *Culex sp*. dynamics and help to reach decisions along the various interventions that can be made needed for public health.

## 2. Materials and Methods

Markov chain model is initially proposed for addressing the problem of predicting the time evolution of mosquito population dynamics throughout the season in a temperate, Mediterranean climate. First, the probability of a population at different population levels, particularly high levels, is predicted; and then the effect of a climate variable, particularly temperature, is included.

### 2.1. Stochastic Process of Ecological Time Series

Let *X*(*t*) be the ecological variable (e.g., mosquito population, or climatic variable), which is considered as a stochastic process that evolves in time t and is defined in a probability space (**Ω**, **F**, ***P***). Where **Ω** is a sample space, **F** is a set of outcomes in the sample space and ***P*** assigns each event of F a probability. If the number of **F** is not countable then the process is denoted by (*X* (*t*)*: t* ≥ 0), or (*Xt*) *t* ≥ 0. In the first case, the process is called a chain in discrete time and in the second, in continuous time. Here the first case is considered, since data have been observed in specific time points and not continuously.

Let **S** be the space created by all possible process values *X*(*t*) in discrete time. If **S** = (**0**, **1**,...) the study refers to a stochastic process with integer values or a discrete state process, e.g., a population threshold or class that corresponds to the number of mosquitos captured in a day, or a class of mean temperature values for that day, etc. Hence, S is considered to take real and finite n values and this contemplative process is called an *n*-dimensional stochastic process.

### 2.2. The Markov Chain Model

The above stochastic process consists of a Markov chain which is determined by its initial state distribution and a transition probability matrix ***P*** of size *m* is [32]:

(1) P(i,j)=Pr[X(t+Δt)=i|(X(t)=j] 1, 2,...,n

The simplest kind of discrete variables the transition matrix may have two stages **S** = (1, 2), which is defined in their simplest form as a high or low level of the ecological variable (e.g., mosquito vector population, temperature) or occurrence or not occurrence (e.g., rain). A sequence of weekly observations constitutes time series of that discrete variable. For the first order Markov chain, the transition probability to future state depends only on its current state. Thus, knowing that at week *i* the variable *X* is either in state 1 (low population levels *X*(*i*) = *a*), or state 2 (high population levels *X*(*i*) = *b*) the related row stochastic transition matrix is:

(2) $$P=\left[p_{ij}\right]=\left[\begin{array}{c} p_{11\;}p_{12\;}\\ p_{21\;}p_{22\;} \end{array}\right]\;\mathrm{Where}\;\{\begin{array}{c} p_{11+}p_{12\;}=1\\ p_{21\;}+p_{22\;}=1 \end{array}$$

By considering m states, **S** = (1,…,*m*) a higher dimension of the transition matrix is formulated as follows:

(3) $$P=\left[p_{ij}\right]=\left[\begin{array}{c} p_{11\;}\\ .\\ .\\ .\\ p_{m1} \end{array}\begin{array}{c} .\\ .\;\;\;\;\\ .\;\;\;\;\;\\ .\\ .\;\; \end{array}\begin{array}{c} .\;\\ .\\ .\\ .\\ . \end{array}\begin{array}{c} p_{1m\;}\\ .\\ .\\ .\\ p_{mm} \end{array}\right]\;\forall i,j=1,\ldots ,m.\;\mathrm{Where}\;\begin{array}{c} \forall i=1,\ldots ,m\\ 0\leq p_{ij}\leq 1\\ \overset{m}{\underset{j=1}{\sum }}p_{ij}=1 \end{array}.$$

A state **S***j* is said accessible from state **S***i* (written **S***i* → **S***j*) if the defined transition system starting in state Si has a positive probability to reach the state **S***j* at a certain point, i.e., ∃*n* > 0: $p_{ij}$ > 0. Two states are said to communicate if both **S***i* → **S***j* and **S***j* → **S***i*. Moreover, any state Si is considered periodic if any return to state Si occur in multiplies of *ki* steps and *ki* is the period and *ki* = GCD (n: ***P****r*(*Xn* = s*i* |*X*_0_ = s*i*) > 0), where *GCD* is the greatest common divisor. Thus, for *ki* = 1 the state Si is aperiodic, else if *ki* > 1 the state Si is periodic with period *ki*. In other words, a state is periodic if after a fixed number of transitions, *ki* > 1, the state can only return it itself otherwise it is aperiodic.

### 2.3. Data Inferred Markov Chain Modeling

Because the knowledge over the time evolution of the current mosquito population process is based on trap captures and is thus limited to derive laws and construct parameterizations from first principles, a data-driven method is used to construct parameterizations by inferring from data. Moreover, the number of adults is considered captured in the CO_2_ traps as a proxy of both, the size of the population and the related mosquito activity levels and despite that different traps vary in their ability to catch certain species.

First, the data are classified into different scale states (e.g., population levels) and a matrix is estimated for each scale state. In particular, for *m* states there have to be *m*^2^ matrix entries to be estimated. The transition probability matrix entry ***P***(*i*,*j*) is estimated as follows (32):

(4) $$\hat{p}\left(m,n\right)=\frac{T\left(i,j\right)}{\Sigma_{j}T\left(i,j\right)}$$

where T(i,j) counting for the transition from m to n observed states observed in a given data set and $\hat{p}\left(i,j\right)$ is the maximum likelihood estimator of *p*(*i*,*j*).

Thus, the Markov chains are “trained with” data from real observations with the aim of mimicking the observed behavior of the population process afterwards in which a finite state MC is inferred from data by estimating its transition probability matrix:

(5) $$T\left(i,j\right)=\underset{t}{\sum }1\left[X\left(t+\Delta t\right)=i\right]1[X\left(t\right)=j]$$

### 2.4. The Conditional Markov Chain Model

The conditional Markov chain (CMC) model is formulated for the case analyzing the occurrence and level of mosquito population depending on the physical state of climate conditions. Particularly, since mosquitos are arthropods and all arthropods are poikilothermic organisms, their development and occurrence of states are affected by temperatures and rain (i.e., favorable versus unfavorable climate). This means that if a Markov chain is used to mimic the process of mosquito population occurrence *X*(*t*), it can be improved by taking into account the condition of a second process *Y*(*t*) which is related to a climate variable (e.g., state transitions of temperature or rain levels). Under this assumption, probabilities take the following form [31,57]:

(6) $$P_{\gamma }\left(i,j\right)=Pr\left[X\left(t+\Delta t\right)=i|(X\left(t\right)=j,Y\left(t\right)=\gamma \right],\;t=1,\;2,\ldots ,n\;$$

### 2.5. Data Inferred Conditional Markov Chain Modeling

If a finite number of states is considered (say five as presented later), then it is possible to construct a CMC model by estimating a transition probability matrix, $P_{\gamma }$, for each possible state. This can be done by knowing the time evolution of the ecological time series for a finite number of states that is used as basis to train the chain model. The transition matrix is estimated as follows [32]:

(7) $$P_{\gamma }\left(i,j\right)=\frac{T_{\gamma }\left(i,j\right)}{\Sigma_{\gamma }T_{\gamma }\left(i,j\right)},$$

In which

(8) $$T_{\gamma }\left(i,j\right)=\sum_{t}1\left[X\left(t+\Delta t\right)=i\right]1\left[X\left(t\right)=j\right]1\left[X\left(t\right)=\gamma \right)]$$

In which **1** is the indicator function: **1**(*A*) = **1** if *A* is true and **1**(*A*) = 0 if *A* is false, while t runs over time instances in the data set used to train the Markov chain model.

### 2.6. Data Encoding and Determination of Transition States

In order to work with finite state conditional Markov chains, vector population dynamics and climatic variables, must be discrete and coded to a finite number of states. If the data are uniformly distributed, this can be done using tree classification schemes based on pre-defined thresholds.

However, since ecological data are most often not uniformly distributed, choosing thresholds is difficult and could result in classes to which no data are assigned and classes to which almost all data are assigned. Moreover, to overcome the problem of subjective defining the different mosquito population levels, k-means clustering algorithm has been used for partitioning the sequence of different population levels in different states based on their centroids.

In particular, each set of mosquito population observations *x1* was considered, *x2*,…,*xn* as a *d*-dimensional real vector and implemented a standard, *k*-means clustering algorithm to partition the n observations into *k* sets (*k* ≤ *n*) that correspond to discrete population states: **S** = (**S**1, **S**2, …, **S***k*) so as to minimize the within-cluster sum of squares (WCSS):

(9) $$arg_{S}\;min\overset{k}{\underset{i=1}{\sum }}\underset{x_{j}}{\sum }{\left\|x_{j}-\mu_{i}\right\|}^{2}$$

where *μ_i_* is the mean of points in *S_i_*.

Let *n_ij_* denote the number of individuals who were in state *i* in period *t* − 1 and are in state *j* in period *t*. The probability of a mosquito population being in state *j* in period *t* given that they were in state *i* in period *t* − 1, denoted by *p_ij_*, can be estimated using the following formula [58]:

(10) $$p_{ij}=\frac{n_{ij}}{\sum_{j}n_{ij}}$$

The probability of transition from any given state *i* is equal to the proportion of mosquitos that started in state *i* and ended in state *j* as a proportion of all individuals in that started in state *i*.

Thus, using the above scheme, the observed behavioral stream of population dynamics was first converted into a symbolic sequence of population states to be used later on the estimation of the transition matrices. It was possible to estimate a transition matrix for each case using mosquito count data.

### 2.7. Markov Chain Model Validation and Equilibrium Distribution

Field data on frequencies of successive mosquito population levels, which were not used for the data inferred MC modeling, were assembled to obtain an empirical intensity transition matrix. Then the empirical transition matrices were generated, and the observed frequencies were compared visually, as well as statistically, with those obtained from the MC models. Two methods were used to evaluate the equidistribution between the observed sequences and that of the MC models.

First, the homogeneity of the transition matrices was tested using a Chi-square (ChiSq) minimum discrimination statistic test. The *verifyHomogeneity* function was applied using the R package makovchain [59,60]. Considering in 2.1. the time evolution of the mosquito population as a stochastic process that generates: *i* = 1, 2, …, *n* discrete time Markov chain samples and that the cardinality of the state space is S the Homogeneity function verifies whether *l* chains belong to the same unknown one. The function shows that its test statistics follows a chi-square law and is estimated as follows (59):

(11) $$2*\sum_{i=1}^{l}\sum_{j=1}^{S}\sum_{k=1}^{S}f_{ijk}ln\frac{n*f_{ijk}}{f_{i\ldots }f_{.jk}}\sim x^{2}\left(r*\left(r-1\right)\right)$$

If there are *l* realizations of a Markov chain of order 1 with *S* states, the null hypothesis, ***H***_0_, is that the transition probability matrix is the same for all *i* and for every possible pairing of *j* and *k* and ***P***(*X* > Chsq), which is less than or equal to the significant level, α = 0.05

In the current work, the case of *l* = 2 chains was considered as two realizations of the **S** = 5-state Markov chains (theoretical and empirical) that are tested for homogeneity. The frequency entries, are: $f_{ijk}$ and *i* = 1,2, *j* = 1,2,… and *k* = 1,2,..,5.

Secondly, the asymptotic distribution properties of the theoretical and the empirical intensity transition matrices were compared using an entropy-based divergence distance measure [54]. In this case, the mosquito population stochastic process was considered as homogenous, and thus starting from an initial distribution of population states a limiting probability, $\Pi_{i}$, exist:

(12) $$\underset{n\to \infty }{\lim}\left(P_{i}\right)=\Pi_{i}{}^{},\;i=1,2,\ldots .,n$$

It follows that:

(13) $$\overset{\infty }{\underset{i=0}{\sum }}\Pi_{i}=1$$

This is called the normalizing condition. Entropy is further associated:

(14) $$H_{i}=-\overset{n}{\underset{j=1}{\sum }}p_{ij}\log p_{ij}$$

$H_{i}$ represents the average amount of uncertainty of the population system for moving one step ahead being initially in state **S***i*. We are now interested in estimating the average uncertainty of the chain for moving one step ahead of any other initial state, which is [54]:

(15) $$H\left(X\right)=H\left(P\right)=-\overset{n}{\underset{i=1}{\sum }}\overset{n}{\underset{j=1}{\sum }}p_{i}p_{ij}\log p_{ij}$$

and consider the Markov chain process as ergodic (e.g., MC and CMC models, as well as the observed mosquito population process used for model validation), so that:

(16) $$H\left(X\right)=\underset{n\to \infty }{\lim}{\left(H^{t}/t\right)}^{}$$

Initially, we define a distance measure by introducing the following norm:

(17) $$\|H_{t}-H_{t=0}\|,\;t=1,\;2,\ldots .$$

where $H_{t=0}$ the entropy associated with the initial probability distribution $\Pi_{i=1}$ representing the different mosquito population levels and $H_{t}$,the entropy of each time step ahead t = 2, 3…

The above scheme quantifies the rate of convergence from a starting non-equilibrium probability distribution towards equilibrium.

### 2.8. Data

We applied the conditional Markov chain model using free mosquito trap data available from the open European Union Data Portal (EU ODP) (http://data.europa.eu, accessed on 3 May 2019), which provides access to data from the European Union (EU) institutions and other EU bodies, which can be reused for commercial or non-commercial purposes (European Commission Decision 2011/833/EU). In particular, we used adult mosquito trap data of *Culex sp*. sampled from 11 locations in central Macedonia–Greece. Data were handled as vectors which consisted of close to weekly time intervals of the number of adult mosquitos captured in CO2 traps from mid-May to September. We used data during two successive observation years (2011 and 2012) for training the Markov chain models, whilst data of one additional year, 2013, were used for validating the model performance.

Because of slight differences between the time intervals of some of the trap counts, data were transformed to mosquito per trap per day (MTD) and thereby averaged over the 11 nearby sampling locations [56]. The MTD thus estimates the average number of mosquitos captured on a day that the trap is exposed in the field.

Weather data and particularly mean air temperatures in Celsius and rain events in mm, were obtained by the national observatory of Athens through a meteorological station, which is located in Makrohori town, which in the same location and latitude and near to the mosquito observation area (http://stratus.meteo.noa.gr/front, accessed on 2 April 2020).

## 3. Results

### 3.1. Mosquito Population Dynamics and the Cross-Correlation with Weather Variables

**Figure 1:**
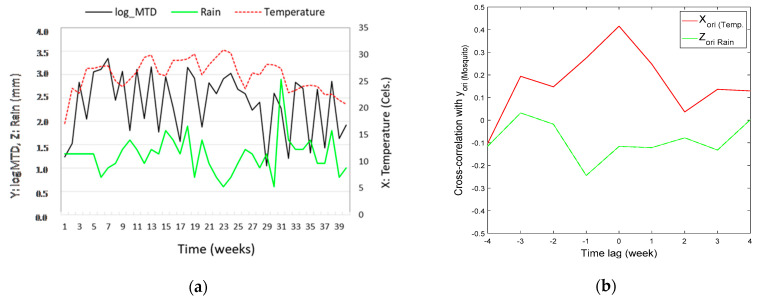
(**a**) Observed time series of mosquito abundance-natural logarithm (Y: log MTD), rain levels (Z: Rain) and mean temperature (X: Temperature (°C)). The data correspond to two consecutive periods of mosquito activity (combined periods of 2011 and 2012). If there are multiple panels, they should be listed as: (**b**) cross-correlation between mosquito abundance and temperature (red line) and between mosquito abundance and rain (green line) as function of time lag (weeks). Note that there is a positive correlation for temperature and that the cross-correlation at time lag zero is highest for temperature. Hence, it is best to condition the temperature at the moment itself and not a week earlier or a week later.

Successive mosquito catches through the observation period and meteorological data are presented in Figure 1. These data correspond to the standardized weekly counts of *Culex* mosquito species (MTD), as well as the mean temperatures (°C) and rainfall incidence (mm) recorded in two successive seasons (2011 and 2012) of typical mosquito activity (May through September) under a typical temperate climate in Northern Greece. As presented in Figure 1a there are similarities in the time evolution of the three variables, but it is not easy to evaluate the degree to which this occurs. Nevertheless, based on the cross-correlation analysis, we determine that it is preferable to use temperature to condition the abundance of arthropod vectors, as it has a much stronger correlation than the impact of rain (Figure 1b). For the cross-correlation analysis all the available data were used in a combined way to detect inherent correlations and increase the validity of the results.

The cross-correlation at time lag zero is highest for temperature (i.e., mosquitos are not lagging temperature), and there is a positive correlation for temperature and a negative relationship for rain. This is in consistent with previous studies [56], suggesting that higher temperatures result in more adult mosquito populations, while the opposite occurs for rain which probably affects flight activity. Based on the time lagged correlation analysis, we derive that it is best to condition on temperature at the moment itself and not temperature a week earlier or a week later. However, we cannot exclude the possibility that in reality mosquitoes could lag the temperature for shorter time intervals (i.e., by maybe one single day), but this cannot be detected since the data consist of averages over a week.

### 3.2. Data Partitioning, Population Transition Networks, and Model Training

After using k-means clustering algorithm, the k cluster centers have been used for positioning the data and considering first a case of two states (high and low levels of population) and then five states (very low, low, intermediate, high and very high population level). The same process may be applied to temperature data. First, we consider a case of two states (high and low temperature levels) and then five states (very low, low, intermediate, high and very high temperature level).

Moreover, because we expected transition probabilities to have been affected by the length of the input data, we performed a preliminary analysis using input variables of different lengths to find the point from which sequence size does not differentiate transition probabilities of the mosquito population. To date, for a sequence length of >35 weeks, the model parameters do not differ considerably according to their informational entropy content and the system explicit a random behavior rather than deterministic suggesting that to a high degree an exact underlying transition matrix exists.

**Figure 2:**
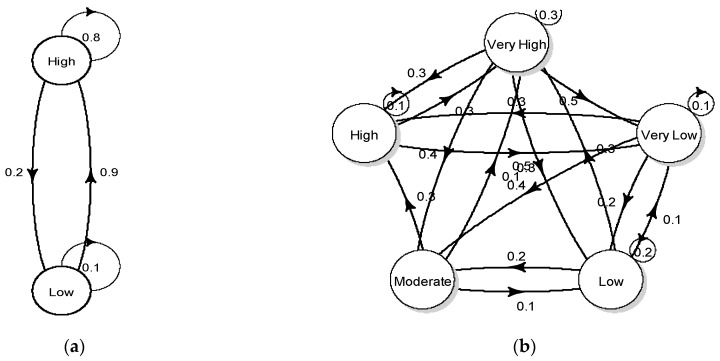
Transition matrix of the mosquito abundance system illustrated in terms of a directed graph (or network). (**a**) The transition matrix has been constructed for two states (high and low) and (**b**) for five stages (very low, low, intermediate, high and very high). Values indicate the probabilities of transitions.

Figure 2 depicts a directed graph (or trained network), which represents the actual transition matrix of the mosquito abundance systems. The criterion of determining how many states to use in the Markov chain depends on both the characteristics of the time series data (range) and the criterion used to limit the classes (e.g., k-means) as previously described. In this work, we decided to use the simplest case, having two clusters as well as the example of having five states based on the data range distribution and k-means clustering statistics. Thus, the transition matrix and the relative graph shown were built for a vector population system with two states (high and low) and five stages (very low, low, intermediate, high and very high) (Figure 2a,b, respectively).

The values that are shown represent the probability of transition from one state to another in the form of an arrow. States are represented as vertices (or nodes), whereas transitions are represented as directed edges (or links). This representation scheme allows the population system of mosquitoes to change from state 1 (e.g., node *i*—low population) to state 2 (e.g., node j high population) along the k edges of the graph through a path of length k from *i* to *j*. For the five states system, for instance, the transition probabilities show that if for a week the mosquito abundance is high (state 4), there is a 10% chance that it will remain at the same level the next week (state 4), 30% chance that it will be at a very high level (state 5), a 30% chance that it will be move to a very low level (state 1) and no chance to move to any of the other population levels (state 2 and 3) (Figure 2b). Yet, it should be noted that the zero probabilities to move to state 2 or state 3 could be an indication that there is some uncertainty of the estimation process related to the particular data set used, since if more data were available, these transition probabilities would be observed with a low probability instead of zero.

### 3.3. Markov Chain Model Realizations of Mosquito Population Levels

**Figure 3:**
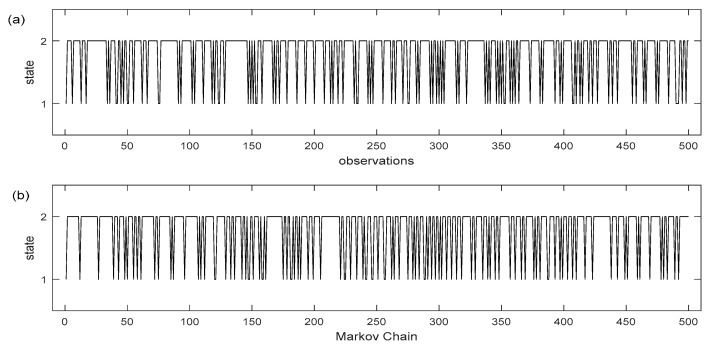
(**a**) Long term sequence of observation of the mosquito population process having two states, (**b**): realization of a Markov chain trained model on these observations, (state 1: low mosquito population, state 2: high mosquito population. Each time period corresponds to an observation for a finite number of successive seasons. The initial state at time 0 corresponds to the first observation made.

**Figure 4:**
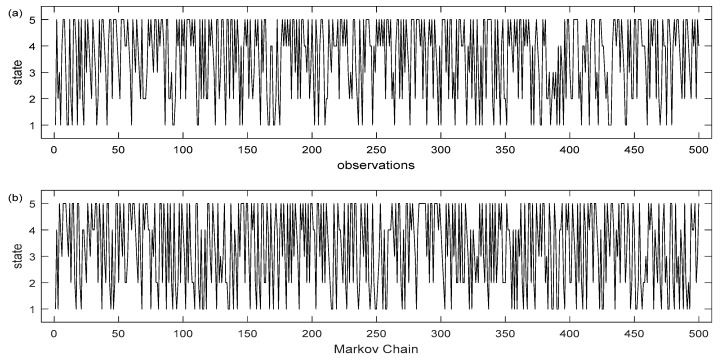
(**a**): Long term sequence of observation of the mosquito population process having five states, (**b**): realization of a Markov chain trained model on these observations, (state 1: very low mosquito population, state 2: low mosquito population, state 3: moderate mosquito population, state 4: high mosquito population, state 5: very high mosquito population). Each time period corresponds to an observation for a finite number of successive seasons. The initial state at time 0 corresponds to the first observation made.

Utilizing the data from the transition matrices, Matlab simulations were executed based on the two and five states Markov chain models, respectively (Figure 3 and Figure 4). The charts show the weekly sequence of the mosquito population levels with two and five states where the stochastic process *X*(*t*) remains in the same state or moves from one state to another, depending on the probabilities of the transition matrix. At each discrete moment of the Markov process or using ecological terms—after each week of observation, a mosquito control decision will be made depending on the predicted population levels. The basis of decision will be the prediction based on how the Markov chain evolves based on the values in the transition matrix P. The division into five population states, compared with only two, results in sections of the series where the *X*(*t*) process presents large deviations from the probability range of values as it evolves over time. By making predictions over time, in order to make decision-making actions, it is our intention to capture the time point where the probability of a high population increases and to avoid any action if the prediction shows that the probability value of the observed variable will decrease. Otherwise, if the forecast indicates that the observed arthropod vector population variable will remain in its present state over a longer period (i.e., low or moderate population level), it is of little practical interest as it is difficult in that situation to decide to undertake control action.

### 3.4. Conditional Markov Chain Model Realizations of Mosquito Population Levels

**Figure 5:**
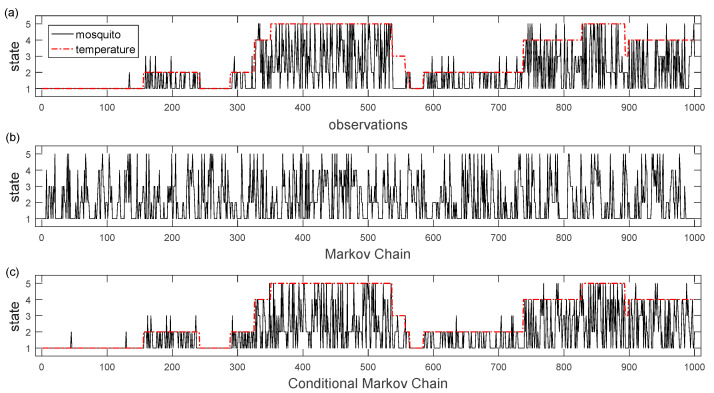
(**a**) Long term sequence of observation of the mosquito population process having five states (straight black line) and the process of a large scale temperature having five states (red dash—dotted line), respectively, and acting as background variable (**b**): realization of a Markov chain trained model on these observations and (**c**) realization of a conditional Markov chain trained on these observations (State 1: very low mosquito population or temperature levels, state 2: low mosquito population or temperature levels, state 3: moderate mosquito population or temperature levels, state 4: high mosquito population or temperature levels, state 5: very high mosquito population or temperature levels). Each time period corresponds to an observation for a finite number of successive seasons. The initial state at time 0 corresponds to the first observation made.

Taking into account the temperature-dependent Markov chain, the situation is different from the previous example because an additional variable is known from the model which can improve simulation performance and population prediction efficiency (Figure 5). Due to the relatively limited data set and to avoid less accurate transition probabilities the CMC model in this study was trained and tested on the same two season’s data. The actual observations that have been used to run the model (Figure 5a) are very close to the realizations generated by the conditional Markov chain model which was trained on these observations (Figure 5c) in contrast to the realization of the non-conditioned of the Markov chain model (Figure 5b). Again, the prediction is built solely on empirical data from the past and never from the future, which is very important to decision-making. Say, for example, that at a given time point, but before making any control action decision, we know the states of the process in the two preceding moments, then we can judge to implement an action against the vector if the population level prediction is high. The simulation results with the conditional Markov chain model we came up with more promising results than those with the simple Markov chain.

### 3.5. Validation of Markov Chain Models and Homogeneity

**Figure 6:**
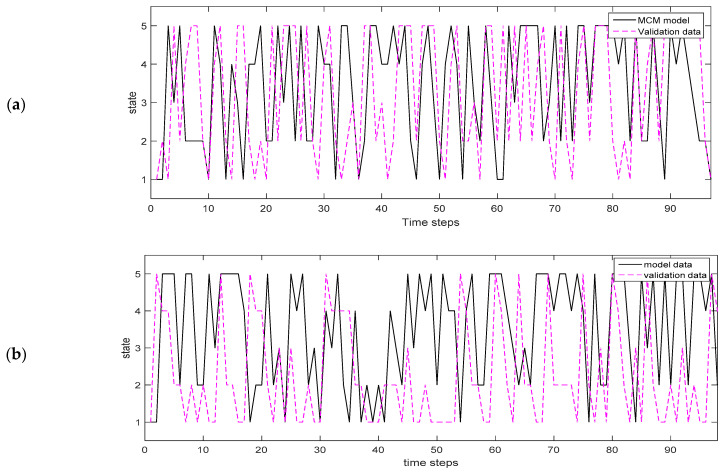
Medium term sequence of observations of the mosquito population process having five states generated from the trained model (black strait line) and that of the empirical intensity transition matrix. (**a**) Markov chain model and (**b**) conditional Markov chain model (pink dashed line) which were resembled using data that were not used for MC model training.

Figure 6a,b depicts the sequence of realizations generated from the trained MC and MCM models and that of the empirical intensity transition matrix, respectively, and which were created using data that were not used for MC model training. The MC model and the empirical realization follow a similar pattern although there are also slight deviations in some time points. These deviances could be justified by the fact that the amount of data available for model evaluation was relatively small. Nevertheless, over all the general model patterns fit well to that of the observed probabilities and this is in accordance with the results of the Chi-square test which test the null hypothesis that both realizations are homogenous, that is, they come from the same matrix of transition probabilities. Particularly, there were no significant differences between the transition matrix of the MC model and the transition matrix used for validation (ChiSq = 18.73683, df = 24, *p* = 0.7658748). Additionally, no significant differences were observed between the CMC model and the validation matrix (ChiSq = 14.58013, df = 24, *p* = 0.9324451).

### 3.6. Limiting Probabilities of Markov Chain Models and Stationary Distribution

**Figure 7:**
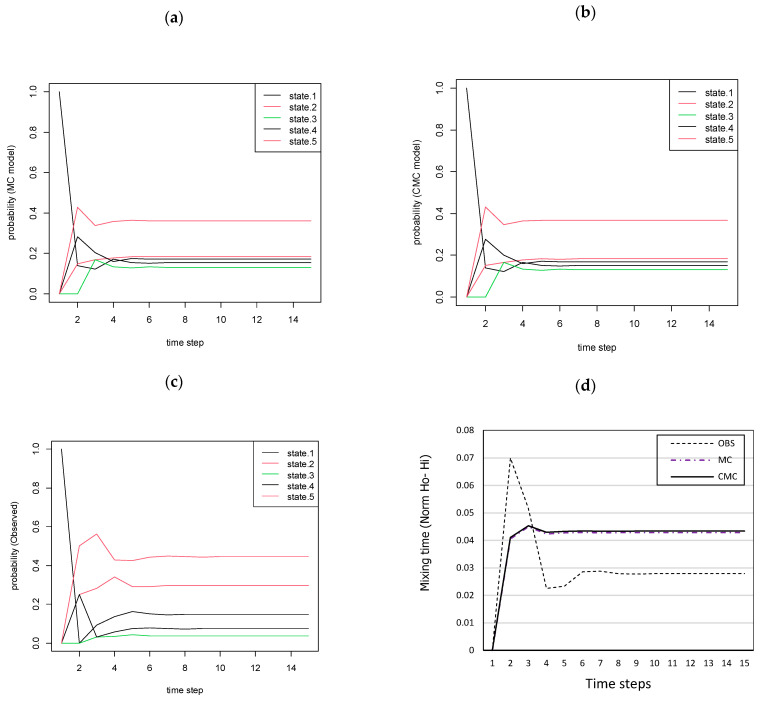
Limiting probabilities of a 5-state Markov chain according to the MC model (**a**), the CMC model (**b**) and the empirical MC of the observed validation data (**c**), as well as the mixing times towards the steady-state probability (**d**). The steady state represents the equilibrium distribution when the mosquito population dynamics is considered as ergodic process.

The convergence of the three Markov chain (MC, CMC models and observed transitions) are illustrated in Figure 7a–c, respectively. Our findings match to certain degree the empirical results in which the probabilities follow analogous patterns. For instance, by looking at the plot we observe that all probabilities convergence fast and the final probabilities are analogous to the stationary distribution. Yet, the chain which was developed using the empirical data shows slight deviations and especially for low population levels, although they finally converge with a consistent rate.

**Figure 8:**
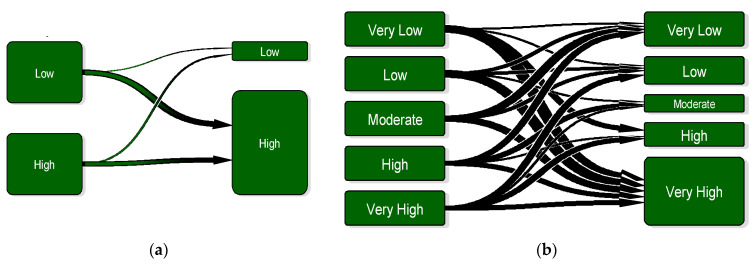
(**a**) Mosquito population transition network with two states and (**b**), mosquito population transition network having five states. Thicker arrows indicate greater probability of transitions.

Figure 8 illustrates the similarities of the time evolution of the entropy, which is related to the average probability of the MC, CMC and the validation data, starting from the initial probabilities of mosquito population levels towards equilibrium (e.g., stationary distribution for each case). The Markov chain models have shown very similar convergence patterns towards equilibrium.

A different representation of the conditional data driven Markov chain transition matrix (e.g., direct graph) is shown in Figure 8 and illustrates of how and to what extent the system evolves after many steps (probabilities are not indicated). Based on this representation, we conclude that it is more likely for the system to go into a state of high population level, when the former is a state of a low population level. On the other hand, there is a lower probability to remain in the same state of low or even high population pressure, especially in the case of the five-state transition matrix. So, the transition matrix can be the foundation for the decision on a control method in a given moment – either a high or moderate arthropod vector population level, depending on the probability of occurrence of a state lying below or above the current level.

## 4. Discussion

Existing and emerging vector-borne diseases are representing one of the most important challenges to public health policy. The novelty of the work consists of the current methodology to simulate, predict the population dynamics of medically important vectors, and the determination for the first time of transition matrices from mosquito field data. This work is first of its kind which apply conditional Markov chains to predict *Culex* sp. adults’ dynamics and considering that most models are of deterministic nature. This approach could be helpful to develop control programs for vector-borne arthropods.

Compared to other models the MCs are simple and thus preferred in modeling complex systems and without detailed knowledge on their function, in order to study their performance and dependability to exogenous factors. However, although the current models have high prediction accuracy, they face the limitation of not providing a strict phenomenological explanation of the system. As a result, a direct biological interpretation of the current model parameters (transitions), as in the case of the Leslie model, cannot be made.

Moreover, the transition values estimated here might be the final result of the environmental conditions that are affecting vital life events of the *Culex* sp. natural populations. In other words, probabilistic models generated are an accurate abstraction of the particular species population process observed in this study and characteristic of the dataset used for their generation. From an entomological standpoint, in order to predict the population patterns of other insect species, the current models should be retrained with population and weather data of the new species and location of interest.

Moreover, it important to clarify that we have used empirical data of *Culex* sp. adults captured in CO_2_ traps which are further considered as a proxy for both, the size of the population, as well as the inherent mosquito activity of the particular study region. However, it is known in entomological studies that due to different modes of actions different traps may vary in their ability to catch certain species [61,62,63,64], while sampling condition such as the location of the trap [65], or the number of nights over which sampling occur [66], as well as weather conditions may also influence trapping results. Nevertheless, despite these limitations, the empirical data used provide means to interpret *Culex* sp. activity that is highly likely to transmit the virus. Actually, variations in trapping outcomes of different data sets may not limit the application and inference of the current models since they deal with probabilities of population level successions rather than forecasting abundances of mosquito individuals *per se*.

Based on the results of this study we conclude that model performance can be improved when temperature is taken into account and this is because environmental fluctuations are translated into population fluctuations through temperature-dependent difference in intrinsic demographic parameters (i.e., survivorship, fecundity, feeding behavior, etc). However, it is important to clarify that in this article we are mainly interested with predictions rather than forecasts despite that we consider the temporal dimension (i.e., weeks). Forecasting, *sensu stricto*, may be considered as a subfield of prediction which is used on the basis of future time series data generation. This is justified by the fact that we fit the model to a training data set, which results in a model that estimates the outcomes for unseen state transitions in terms of probabilities, rather than estimates of the actual mosquito abundance values (i.e., time series). Moreover, a similar approach has been applied on the validation data set to generate a future sequence of mosquito population states to be compared with the model predictions. This was also the main reason why we have used the Square-test to compare transition matrices, as well as related information measures, such as Shannon entropy, to compare the time evolution of the models towards equilibrium, rather than a correlation analysis.

Summarizing the modeling approach and related simulations, we consider here to look at some of the features of the conditional Markov chain modelling method under consideration. Classical conditional Markov chain models (also known as linear-chain conditional random fields in the literature e.g., Lafferty et al. 1999 [57]), were defined by Bielecki and Rutkowski in 2004 [55], for applications in finance and insurance. Conditional Markov chains, as proposed in the current work, have been also used in atmospheric science [32]. This is done in response to the need for modeling dependence between dynamic systems in cases when some conditional properties of a system are important and should be accounted for. Hence, conditional Markov chains are defined as a versatile class of discriminative models for the distribution of a sequence of hidden or latent states conditional on a sequence of observable variables.

However, it should be noted that although the concept of Markov chains has been used already in pioneering works of theoretical biology [51,52,53], in this work it is applied under a different context. For example, classical works in the field of transition models deal with age-structured cohorts, where survivorship and net maternity is known and extend the already know Leslie model to describe the limiting behavior of population growth and its sensitivity to environmental perturbations. Here, the use of Markov chains differs conceptually compared to classical theoretical Leslie projection schemes, since we have no prior information of the initial stage specific population structure and its demographic characteristics, and the only available information is the adult abundances which were estimated by traps. Contrarily, this work aims first on the partition of data, using a classical clustering algorithm, and later predicting the transitions of different mosquito population levels forced by temperature. Thus, this empirical work emphasizes applied modeling of a populations data sequence, which is most often available in entomological surveys, rather than a theoretical study which focus on a complete characterization of the species life-cycle transitions as a result of births and deaths.

Based on the simulation results it is apparent the model performs better when it is conditioned on temperature. In accordance with other studies, this work shows that environmental changes have impacts on demographic parameters and are reflected in the species population. In the case of the observed and predicted *Culex sp*. dynamics, it can be argued that temperature alteration during the season induce changes in developmental rates, survivorship and net maternity could underlie the transitions between the different population states.

In that sense our model simulations might suggest that a persistence of increased temperature levels for longer periods could be linked with an increase in fecundity and survival of immature stages, suggesting an increased fitness which is reflected to a sequence of high *Culex sp*. population levels. In the same sense, one cannot exclude the opposite function when temperature levels are lower. Moreover, other factors, which have not considered here, such as wind, relative humidity, could also affect *Culex sp*. feeding behavior and related dynamics. All these factors may be essential for the eco-biology of the species and affect its observed dynamics.

Given the importance of climate conditions for mosquito development, especially temperature and rain, it is necessary to take into account these variables in predicting mosquito populations. Furthermore, the estimation of the transition matrix through the use of empirical data first to define the system states and later on for training and validating the Markov chain model is a principal step for the simulation of realistic vector population projections and without the need for defining differential equations and related state variables. The CMC model that is proposed in this study might prove very suitable in public health decision making and especially for predicting arthropod vector population dynamic and vector controls. Several mathematical models have been developed to clarify and predict the dynamics of mosquito populations and to understand the role of environmental factors [67,68,69,70]. In most studies mosquito population dynamics are treated as deterministic processes (among others [71,72]) despite populations being driven by climate factors which are considered to be probabilistic [73,74,75,76,77]. Therefore, it is difficult to perform a direct comparison of our modeling approach and results, to other related studies, since most of them are based on deterministic-dynamic population models. However, multivariate ecological and epidemiological time series are characterized by complex non-linear relationships and most often explicit a random behavior [78].

Hence, the proposed MC stochastic model provides a robust alternative to traditional models. For instance, deterministic models cannot capture population fluctuations which are dominated by environmental conditions, variability in the controlling parameters as well as the random nature of population events, which occur in a real system [33]. Moreover, the fact that the projection of mosquito population dynamics is improved by incorporation of information on temperature levels in terms of conditional probabilities is in accordance with most studies that acknowledge the significance of climate factors and temperatures particularly in arthropod vector population dynamics and related disease epidemics [78,79,80].

Based on the current cross correlation results, we conclude that temperature exert a higher impact on the *Culex sp*. adult phenology compared to rain events, despite mosquitos thriving in wet conditions as rain indirectly affects the mosquito population by increasing breeding grounds. Therefore, it was judged as necessary to include the most influential meteorological variables (e.g., only temperature) to improve the performance of a simple MC model through the use of a CMC model instead. Actually, it was found that Markov chain model of arthropod vector population dynamics, conditioned over temperatures, performed better than single MC stochastic modeling of vector population dynamics.

After the importance of the meteorological conditions was found, it became apparent that once the population reaches a high state during a week, there is a very high probability that it will remain in this state the following week and so on. Moreover, considering that time evolution of temperature states is quite analogous of that of the arthropod vector states, we can conclude that if there is a high probability of increased temperatures, we expect an increased probability to observe very high mosquito levels. Thus, a part of the CMC mode results, modeling only temperatures through MC model may be proved very useful in judging whether during the same period the mosquito population is also high. This is of major importance for public health management and vector eradication programs considering saving costs and time for the establishment of mosquito surveillance programs over different areas. Thus, this information becomes crucial for preventing the transmission of mosquito disease prioritizing resources for optimal responses in vector eradication programs and mosquito surveillance programs.

Considering model validation, the transition probabilities of both Markov chain models (e.g., the simple, as well as the conditional one) do not differ significantly to the empirical data. However, there should also be some wariness as the data set used for model validation, despite being representative for a mosquito activity season, was only from a year of observations. Nevertheless, overall, the MC model and the empirical realization follow a similar pattern although despite some expected deviations.

The presented results are promising, although we stress that they were obtained under certain assumptions, such as the stability of the particular study environment and the conditioning over only one climate variable. For instance, Markov models are generally inappropriate over sufficiently short sequence lengths and time intervals yielding in a process which is deterministically related to time rather than random to resolve this problem we decided to evaluate the effect of different sequence lengths on the informational content of different MC sample trajectories. Additionally, considering the data sampling intervals of mosquito abundances, we have decided to use normalized weekly counts, for both, model training as well as for the predictions, since these are most often used in entomological studies to capture the dynamics of ecological processes.

In reality, additional ecological factors which have not been taken into account in this study may affect mosquito population dynamics in a more complex manner. Among such factors is the possibility of a parallel influence of two or more climate variables on the mosquito population dynamics or even a more substantial influence of lagged values that suggested also testing the model performance of a higher order Markov chain model.

Moreover, to reach more realistic Markov chain projection schemes, they should probably be compared and trained with additional observational data. Nevertheless, the current work outlines how the Markov chain models can be applied in ecological time series and particularly in modeling arthropod vector dynamics. Furthermore, the current work contributes to recent tendencies in ecological modeling which focus on the integration of climate factors and related weather variables in functioning of population processes. Another future direction which may be worth verifying would be the calculation of a multivariate semi-Markov conditional model with different orders.

## 5. Conclusions

This work introduces a new stochastic mathematical model for modeling the population of arthropod vectors (the world’s deadliest animal, which accounts for 80% of human vector-borne diseases) applied in predicting *Culex* sp. abundances. Conceptually, the insect captures are treated as a random sequence of different population levels that evolve in time and are characterized by the Markov property. Compared to traditional modeling approaches in entomology the current approach is desirable because it may allow reasoning and resolution of problems and complex population systems with little knowledge of its internal workings. The most important asset of the current research is the evolution of a model which potentially can be applied as a method for detecting and forecasting the periods in which there is a high probability of vector population persistence. The results show that conditional modeling of the Markov chain is useful for simulating future population dynamics of arthropod vectors. In the context of prevention methods to mitigate the effects of arthropod vector dynamics on public health there is an urgent demand for warning systems to aid decision making. The current model results could form the basis to forecast the probability of “high” and “very high” arthropod vector population levels to alert people belonging to vulnerable groups and to implement effective vector control measures to protect public health during periods of high vector population. Finally, the current subject field can be seen as valuable since it is not just the first of its sort in modeling *Culex* sp., but proposes novel methods that encourage further modeling and exploration of the behavior of more complex vector population systems.

## Data Availability

Publically available datasets were analyzed in this study. This data can be found here: [http://data.europa.eu (accessed on 3 May 2019)] and here: [http://stratus.meteo.noa.gr/front (accessed on 2 April 2020)].
